# Community pharmacists’ knowledge and practices towards antimicrobial stewardship: findings and implications

**DOI:** 10.1093/jacamr/dlae176

**Published:** 2024-10-29

**Authors:** Webrod Mufwambi, Kunda Musuku, Jimmy Hangoma, Ngoni Veddie Muzondo, Larry Mweetwa, Steward Mudenda

**Affiliations:** Department of Pharmacy, School of Health Sciences, University of Zambia, P.O. Box 50110, Lusaka, Zambia; Department of Pharmacy, School of Health Sciences, University of Zambia, P.O. Box 50110, Lusaka, Zambia; Department of Pharmacy, School of Health Sciences, Levy Mwanawasa Medical University, Lusaka, Zambia; Department of Pharmacy, Harare Institute of Technology, Ganges Road, P. O. Box BE 277, Belvedere, Harare, Zimbabwe; Department of Science and Technology, Ministry of Technology and Science, Maxwell House, Los Angeles Boulevard, P. O. Box 50464, Lusaka, Zambia; Department of Pharmacy, School of Health Sciences, University of Zambia, P.O. Box 50110, Lusaka, Zambia

## Abstract

**Background:**

Antimicrobial stewardship (AMS) programmes have been implemented around the world to optimally manage antimicrobial use to attenuate antimicrobial resistance (AMR). This study assessed the knowledge and practices of community pharmacists towards AMS strategies in the Lusaka District, Zambia.

**Methods:**

This cross-sectional study was conducted among 194 community pharmacists in the Lusaka district using a structured questionnaire from August 2022 to September 2022. Data analysis was done using Statistical Package for Social Science (SPSS) version 22.0.

**Results:**

Of the 194 participants, 86% of the community pharmacists had good knowledge of AMS. The present study found that 83.5% were aware of AMS strategies used in community pharmacy practice. Further, 83.5% of the CPs were familiar with the goals of AMS and 89.2% believed that AMS was necessary for their pharmacy practice. Furthermore, 66.7% of the participants exhibited good practice towards the AMS strategies. Finally, 61.3% frequently avoided the use of broad-spectrum antimicrobials that were not necessary.

**Conclusions:**

According to the findings of this study, the majority of community pharmacists had good knowledge of the AMS strategies that were employed to combat AMR; nonetheless, some of them had poor practices. Therefore, there is a need for community pharmacists to have access to sufficient ongoing professional development programmes and educational activities through AMS programmes to address AMR.

## Introduction

Antimicrobial resistance (AMR) is an increasing global public health issue.^1–3^ The emergence and spread of AMR have been linked to pathogen exposure to antimicrobials, with inappropriate use being a major contributing factor.^4–7^ The access to antibiotics without prescriptions is a huge problem that has been linked to the emergence and spread of AMR.^8–11^ Antibiotic-resistant infections are associated with increased morbidity and mortality. Unfortunately, if factors that contribute to AMR are not addressed, then the mortality rate will increase to more than 10 million people annually by the year 2025.^12^

Antimicrobial stewardship (AMS) programmes have been implemented to reduce the inappropriate prescription and use of antimicrobials, improve clinical outcomes, reduce the emergence of AMR and conserve healthcare resources.^13–17^ AMS programmes aim to optimize individual patient outcomes whilst minimizing unintended consequences, such as the generation of drug-resistant pathogens.^18–23^ These programmes involve the use of evidence-based guidelines and educational programmes, and regular feedback on antimicrobial use (AMU) data to prescribers to promote rational and evidence-based prescribing of antimicrobials.^17,24,25^ With AMR on the rise worldwide and few new agents in development, AMS programmes are more important than ever in ensuring the continued efficacy of available antimicrobials.^26^

Evidence has shown that AMS programmes are effective in improving clinical outcomes associated with antimicrobial therapies while improving patient safety by minimizing adverse events and the emergence of AMR.^25,27,28^ Identifying and developing strategies to address barriers and challenges to AMS can facilitate the establishment of financial, administrative and organizational support, and agreement and participation by individual prescribers.^28^ AMS programmes also involve antibiotic policies, antibiotic management and control programmes that promote the rational use of antibiotics.^29^

Pharmacists have an established role as promoters of evidence-based medicine and cost-effective prescribing.^30^ Against a background of increasing AMR, it has been suggested that enhanced pharmacy activities may help to optimize treatment, improve outcomes, promote rational prescribing, reduce inappropriate use and potentially slow the development and spread of resistance.^31–33^ Many programmes initiated as cost-saving measures have put pharmacists at the forefront of many AMS programmes as they act as the effector arms for AMS programmes^34–39^ Although pharmacists are well positioned for these programmes, some may not have adequate training in infectious diseases to feel comfortable providing recommendations for complex cases.^40^ Thus, having a pharmacist with specialized training in infectious diseases dedicated to implementing an AMS programme is important.^26^

Formal training programmes in infectious diseases for pharmacists are expanding and becoming increasingly standardized.^41,42^ Pharmacists are involved in many activities including the development of guidelines for antimicrobial use, educating other healthcare professionals, review of antimicrobial orders with feedback to providers, administration of restrictive strategies, pharmacokinetic consultation and research on programme outcomes.^26^ Community pharmacists (CPs) play pivotal roles in combatting AMR and are critical members of the AMS programmes.^43,44^ Evidence has demonstrated that CPs are aware and have good perceptions regarding AMS programmes.^45,46^ The involvement of CPs in AMS programmes improves antibiotic stewardship and dissemination of information about antibiotics leading to improved patient outcomes.^47,48^ CPs educate and counsel patients about appropriate antibiotic use and collaborate with other healthcare workers to improve patient outcomes.^49^ Nevertheless, there is still a need to promote their roles and involvement in AMS activities in their community practice.^45^

Zambia is a country in sub-Saharan Africa with varying reports on AMR of various pathogens across humans, animals and the environment.^50–59^ Additionally, access to antibiotics without a prescription is quite high in Zambia.^9,10,60–63^ Zambian CPs receive new prescription orders from prescribers and perform identification, evaluation, interpretation, clarification and verification of a prescription before release to the patient.^64^ Further, CPs consult with patients regarding prescription or non-prescription drugs.^64^ Furthermore, they perform professional consultations with other healthcare workers and educate patients about their condition and medicines. Zambian pharmacists select drug products to dispense to the patient.^64^ However, limited information has been documented on the knowledge and practices of CPs regarding AMS strategies in Zambia. Therefore, this study assessed the CPs’ knowledge and practices towards AMS strategies in Lusaka, Zambia.

## Materials and methods

### Study design, site and population

The CPs in the Lusaka area of Zambia participated in this cross-sectional study carried out between the months of August and September 2022. Community pharmacies that were eligible for the study were registered with the Zambia Medicines Regulatory Authority (ZAMRA) in the Lusaka District of Zambia. To be eligible for the study, all CPs needed to be officially registered with the Health Professions Council of Zambia (HPCZ) and provide informed and written consent.

### Sample size estimation and sampling method

To determine the sample size, we used Taro Yamane’s formula.^65^ A total of 370 registered community pharmacies in Lusaka were obtained from the ZAMRA website.^66^ We calculated a minimum sample size of 193 at a 5% margin of error, and we took into anticipation a 10% incomplete or non-response rate. Based on the sample size that was calculated through the use of community pharmacies, a minimum of 193 CPs were targeted. We then listed the registered community pharmacies in Lusaka District, Zambia. A basic simple random sampling procedure based on computer-generated random numbers was utilized to collect data from the CPs. We printed and distributed 213 questionnaires across community pharmacies in the Lusaka District, Zambia. Of the 213 questionnaires distributed, 194 were filled in and included in the analysis.

### Data collection tool

Data collection was done using a structured questionnaire that was adopted from previous studies.^67–69^ The questionnaire was divided into three sections: Section A, which focused on socio-demographic information; Section B, which inquired about the participants’ knowledge of AMS approaches; and Section C, which requested information about the participants’ practice of AMS strategies. Regarding the knowledge variable, a score of 1 was assigned for a correct answer while a score of 0 was assigned to an incorrect answer. About the practice variable, the responses were assigned scores as follows: never = 0, rarely = 1, often = 2 and always = 3. A maximum of 10 points (100%) were awarded for awareness, while a maximum of 24 points (100%) were awarded for practice. Therefore, CPs who scored 80% (8/10) or higher were considered to be aware of AMS. Additionally, CPs who scored 80% (19.2/24) or more were considered to have good practice with AMS. The cut-off point used in this study has been used in previous studies regarding AMS.^40,61,70,71^

### Data analysis

The data gathered were entered into a spreadsheet created in Microsoft Excel (2013), where they were validated twice to ensure that the data were complete. After that, the data were exported to IBM Statistical Package for Social Sciences (SPSS) version 22.0 for descriptive analysis. The analysed data were presented in the form of tables.

### Ethical approval

We obtained ethical approval from the University of Zambia Health Sciences Research Ethics Committee (UNZAHSREC) before the study was carried out, with a protocol authorization number 2022112301238. The purpose of the study was explained to the study participants. Participation in the study was entirely voluntary, and participants were required to provide both informed and written consent.

## Results

Of the 213 distributed questionnaires, 194 were filled in giving a response rate of 91.1%%. Therefore, a total of 194 CPs participated in this study, with 58.8% (*n* = 114) of them being male. Additionally, 57.7% of the participants were aged between 20 and 29 years. In terms of educational attainment, 88.1% (*n* = 171) of the participants held a Bachelor of Pharmacy degree only, while 11.9% (*n* = 23) held a Bachelor of Pharmacy and Master’s degree. Of the 194 participants, 66% (*n* = 128) had 1–5 years of job experience (Table 1).

**Table 1.** Distribution of participant’s socio-demographics

Variable	Response	% (*n*)
Gender	Male	58.8 (114)
	Female	41.2 (80)
	Total	100 (194)
Age (years)	20–29	57.7 (112)
	30–39	26.3 (51)
	40–49	14.4 (28)
	Above 50	1.5 (3)
	Total	100 (194)
Education level	Bachelor’s degree	88.1 (171)
	Bachelor’s and Master’s degree	11.9 (23)
	Total	100 (194)
Work experience (years)	1–5	66 (128)
	6–10	21.65 (42)
	11–15	10.3 (20)
	Above 16	2.1 (4)
	Total	100 (194)

### CPs’ knowledge of AMS

This study found that 85.6% of the CPs were aware of the meaning of AMS, 83.5% were familiar with the goals of AMS, 89.2% believed that AMS was necessary for their pharmacy practice, and 88.1% were aware that AMS involved the appropriate selection of antimicrobials. Further, 88.7% were aware that AMS involved optimal antimicrobial administration, 89.7% agreed that AMS interventions improve patient outcomes, and 88.1% agreed that AMS strategies could reduce the problem of AMR. Furthermore, 85.6% agreed that AMS reduce the length of hospital stay, 80.4% were aware of where to find information on AMS strategies, and 85.6% of the CPs believed that additional staff education on AMS was required (Table 2). Overall, 86% of the CPs had good knowledge regarding AMS programmes.

**Table 2.** Participant’s knowledge of antimicrobial stewardship

Awareness questions	Yes % (*n*)	No % (*n*)	Total
Do you know what AMS means?	85.6 (166)	14.4 (28)	100 (194)
Are you familiar with AMS goals?	83.5 (162)	16.5 (32)	100 (194)
Is AMS essential in this pharmacy?	89.2 (173)	10.8 (21)	100 (194)
Does AMS involve the appropriate selection of antimicrobials?	88.1 (171)	11.9 (23)	100 (194)
Does AMS involve optimal antimicrobial administration?	88.7 (172)	11 (22)	100 (194)
Can AMS interventions improve patient outcomes?	89.7 (174)	10.3 (20)	100 (194)
Can AMS strategies reduce the problem of AMR?	88.1 (171)	11.9 (23)	100 (194)
Can AMS reduce the length of hospital stay?	85.6 (166)	14.4 (28)	100 (194)
Are you aware of the source of information on AMS strategies?	80.4 (156)	19.6 (38)	100 (194)
Is additional staff education on AMS needed?	85.6 (166)	14.4 (28)	100 (194)

### CPs’ practices towards AMS

According to the findings of this study, 27.8% of participants always followed standard treatment guidelines, 61.3% frequently avoided the use of broad-spectrum antimicrobials that were not necessary, 66.5% frequently documented the use of antimicrobials in patient care, and 63.4% consistently audited and reviewed antimicrobial prescriptions. Further, 74.2% of the CPs always educated patients on AMU, 65.5% of the CPs frequently evaluated the use of antimicrobials (both in terms of quality and quantity), and 47.9% of the CPs frequently examined the outcomes of AMU. Furthermore, 58.2% of the CPs had never utilized hospital antimicrobial data (Table 3). Overall, 67% of the CPs had good practices towards AMS programmes.

**Table 3.** Community pharmacists’ practice towards antimicrobial stewardship

Practice questions	Response % (*n*)				
	Never	Rarely	Often	Always	Total
Do you use standard treatment guidelines?	1 (2)	8.2 (16)	62.9 (122)	27.8 (54)	100 (194)
Do you avoid unnecessary broad-spectrum antimicrobial use?	0.5 1	3.1 (6)	61.3 (119)	35.1 (68)	100 (194)
Do you document antimicrobial use in patient care?	2.6 (5)	8.8 (17)	66.5 (129)	22.2 (43)	100 (194)
Do you audit and review antimicrobial prescriptions?	1 (2)	8.8 (17)	26.8 (52)	63.4 (123)	100 (194)
Do you educate patients on antimicrobial use?	0 (0)	10.8 (21)	14.9 (29)	74.2 (144)	100 (194)
Do you assess the antimicrobial use (quality and quantity)?	0.5 (1)	13.4 (26)	65.5 (127)	20.6 (40)	100 (194)
Do you measure the antimicrobial use outcomes?	2.6 (5)	39.7 (77)	47.9 (93)	9.8 (19)	100 (194)
Do you use hospital antimicrobial data?	58.2 (113)	24.7 (48)	13.4 (26)	3.6 (7)	100 (194)

## Discussion

This study assessed the CPs’ knowledge and practices towards AMS strategies in Lusaka, Zambia. Our study found that the CPs were knowledgeable about AMS programmes but their practice did not reflect this good knowledge.

The results of this study revealed that 86% of CPs had good knowledge of the AMS strategies. A previous study conducted in Zambia reported that most healthcare workers tend to have good knowledge of AMR and AMS.^72^ The results of this current study conform with other studies in which pharmacists were reported to have good knowledge of AMS.^69,73,74^ The similarities observed in this current study to other studies can be attributed to the knowledge exhibited due to the knowledge they acquire during training and the experience they attain during their practice.

In this current study, the majority of CPs knew the meaning of AMS. The findings are similar to a study conducted in Australia in which most CPs knew the term AMS, although 75% of them reported an improved understanding after reading the provided definition.^75^ This similarity can be alluded to by the fact that pharmacists are exposed to a lot of information related to antimicrobials in their practice and also due to the information they obtain during their training. However, only 57.3% of the CPs were familiar with the term AMS in Pakistan due to a lack of adequate training on AMS programmes and activities.^76^

The present study revealed that most CPs were familiar with the goals of AMS and 89.2% agreed that AMS is essential in their community pharmacy practice. The findings of this study are in line with those reported in a study conducted in the United Arab Emirates (UAE) in which the CPs recognized the importance of AMS in their facilities as the majority were in agreement that AMS is essential as it improves patient care.^77^ Most CPs tend to understand the goals of AMS programmes including knowing that AMS improve patient outcomes and reduces the inappropriate use of antibiotics.^45,46,77^ However, a study by Erku in Ethiopia reported contrasting findings that revealed that only 26.5% of respondents agreed that AMS should be practised at the community pharmacy level.^78^

AMS strategies cover many education subjects including proper antimicrobial selection and prescription, optimizing doses and duration and minimizing toxicity and side effects to improve clinical, economic and microbiological results.^79^ In this study, most CPs reported that they were involved in the appropriate selection of antimicrobials, 88.7% agreed to AMS involving optimal antimicrobials administration, and 89.7%were aware of AMS interventions in improving patient outcomes. The results in this study are similar to the findings of other researchers in which the majority of respondents strongly agreed or agreed that the AMS programme is vital for the improvement of patient care.^38,78,80^ This similarity can be attributed to high academic training, like having a Bachelor’s or Master’s degree as well as work experience.

The present study found that the majority of CPs agreed that AMS strategies can reduce the problem of AMR and hospital stay. The present study further found that CPs were aware of the source of information on AMS strategies and accepted that additional staff education on AMS was needed. The findings of the current study are in line with those reported in another study where most respondents perceived that AMS programmes could reduce the problem of AMR by promoting the rational use of antimicrobials and improving patient outcomes.^38^ By so doing, AMS in turn reduces the length of hospital stay.^81^ Evidence has shown that there is a need to provide adequate training to CPs on AMU to enhance their understanding of AMS.^45,82^ Many sources of information for AMS education and interventions have been outlined and leveraged to promote AMS in community pharmacies.^83^

This study found that CPs scored an overall 67% concerning practice towards AMS, indicating that the overall practice of CPs regarding AMS was not a reflection of their good knowledge of AMS. This finding is similar to a previous study conducted in Zambia, where most CPs had good knowledge but low AMS practices concerning AMS.^84^ Additionally, a study in Nigeria also reported that despite most of the CPs having good knowledge of AMR and AMS, their practices were poor.^85^ These gaps indicate the need to instigate and strengthen AMS programmes in community pharmacy practice.^34,37,44,86,87^

The present study revealed that the majority of the CPs used STGs during their practice. These findings of our study are similar to those reported in Uganda in which 66.7% of the pharmacists used the treatment guidelines in their practice.^67^ The similarity in the high use of treatment guidelines observed in this current study could be attributed to the care exhibited by most pharmacists in ensuring that good services are delivered to the clients/patients. However, the current study findings contradict those reported in Australia by Saha *et al*.^38^ in which only 45.5% used the national treatment guidelines. Our study also found that the majority of CPs often avoided unnecessary broad-spectrum antimicrobial use. This study also contradicts the findings of another study where only 20.8% avoided unnecessary broad-spectrum antibacterial use.^67^ Pharmacists are greatly positioned to act as an effector arm for AMS programmes because of their responsibility for processing medication orders.^80^ In this current study, most CPs often documented antimicrobial use in patient care, always audited and reviewed antimicrobial prescriptions and always educated patients on antimicrobial use. Our results are in line with the findings of another study in which 70.8% documented antibacterial use, 70.8% audited and reviewed antimicrobial prescriptions and 75% educated patients on the use of antimicrobials and resistance-related issues to prevent or reduce the transmission of infections within the community and informed the public about the importance of following the recommended duration and dosage of antibiotic treatment.^88^ The good practice observed in this study of reviewing and auditing prescriptions, patient education and documentation of antimicrobial use could be attributed to factors such as good knowledge of the importance of documentation as well as the mandate given by the regulatory authority (ZAMRA) in ensuring documentation of every prescription.

Our study revealed that most CPs often assessed the quality and quantity (AMU) but only an average of them often measured the AMU outcomes. The use of antimicrobials may result in the intended therapeutic effect or consequences including its contribution to AMR development, toxicity, increased morbidity and mortality.^89^ Hence, it is important for CPs to assess the consumption of antimicrobials and the possible outcomes. Lastly, most of the CPs did not use hospital antimicrobial data in their practice. The results of this study are similar to the findings of another study where only 25% of CPs used hospital antibacterial audit data.^67^ Auditing the prescribed antibacterials is critical in understanding the prescribing patterns of antibiotics that can affect patient outcomes. These practices also foster collaborations between CPs and prescribers in the interest of the patient.^46,90^

We are aware of the limitations of this study. This study was conducted in the Lusaka District of Zambia, therefore, the findings cannot be generalized to all the 116 districts of the country. We used a cross-sectional study design that may have some bias from participants when responding to the questions. However, our study provides insights into the knowledge and practices of CPs regarding AMS in Lusaka, Zambia.

The findings of this study indicate the need to develop and implement AMS programmes in community pharmacies in Lusaka, Zambia. Further, there is a need to promote continuous professional development education involving rational dispensing and use of antimicrobials, AMR and AMR programmes. The government should extend AMS programmes to community pharmacies through the National Public Health Institute and the Medicines Regulatory Authority.

### Conclusion

This study revealed that, even though CPs had a high level of awareness regarding AMS, their practices were slightly lower. Hence, this can affect their provision of patient care in community pharmacies. Consequently, there is a requirement for the provision of educational intervention activities to promote the rational use of antibiotics in community pharmacies. Last but not least, to enhance and maintain the rational utilization of antibiotics, it is necessary to design and implement AMS programmes in all community pharmacies in Zambia.
